# Selection of Reference Genes for Quantitative Real-Time PCR during Flower Development in Tree Peony (*Paeonia suffruticosa* Andr.)

**DOI:** 10.3389/fpls.2016.00516

**Published:** 2016-04-21

**Authors:** Jian Li, Jigang Han, Yonghong Hu, Ji Yang

**Affiliations:** ^1^Ministry of Education Key Laboratory for Biodiversity Science and Ecological Engineering, Fudan UniversityShanghai, China; ^2^Shanghai Key Laboratory of Plant Functional Genomics and Resources, Shanghai Chenshan Plant Science Research Center, the Chinese Academy of Science, Shanghai Chenshan Botanical GardenShanghai, China

**Keywords:** tree peony, reference genes, gene expression normalization, qRT-PCR, flower development

## Abstract

Tree peony (*Paeonia suffruticosa*) is a perennial plant indigenous to China known for its elegant and vibrantly colorful flowers. A few genes involved in petal pigmentation have been cloned in tree peony. However, to date, there have been few studies on the comparison and selection of stable reference genes for gene expression analysis by quantitative reverse-transcription PCR (qRT-PCR) in this species. In this study, 10 candidate reference genes were evaluated for the normalization of qRT-PCR in three tree peony cultivars. *GAPDH* and *UBC* were identified as the top two most stable reference genes in ‘Feng Dan’ and ‘Xi Shi,’ and *EF-1*α/*UBC* was recommended to be the best combination for ‘Que Hao.’ The expression stability of various reference genes differed across cultivars, suggesting that selection and validation of reliable reference genes for quantitative gene expression analysis was necessary not only for different species but also for different cultivars. The results provided a list of reference genes for further study on gene expression in *P. suffruticosa*. However, in any case, a preliminary check on the accuracy of the best performing reference genes is requested for each qRT-PCR experiment.

## Introduction

Gene expression analysis provides improved understanding and insights into the molecular basis underpinning various biological processes (Bustin et al., 2005). Quantitative real-time polymerase chain reaction (qRT-PCR) is one frequently used platform to quantify transcript abundance, for its sensitivity, accuracy and reproducibility (Gachon et al., 2004). However, the accuracy of qRT-PCR is heavily dependent on the stability of the internal reference genes used to normalize transcript abundances (Huggett et al., 2005). An ideal reference gene must be expressed steadily both in different tissues and at different developmental stages and remain unaffected by any experimental treatment (Czechowski et al., 2005). Many studies have confirmed the fact that the utilization of unstable reference genes may result in significant biases and misinterpretations of data (Ferguson et al., 2010; Mafra et al., 2012). It is thus critical to select suitable reference genes before quantifying the expression level via qRT-PCR.

Various software packages, most prominently geNorm (Vandesompele et al., 2002), NormFinder (Andersen et al., 2004), Bestkeeper (Pfaffl et al., 2004), and RefFinder (Xie et al., 2012) have been developed to identify appropriate reference genes in different experimental systems. They have been widely used in different plants, including the model plant Arabidopsis (Wang H. B. et al., 2014), ornamental plants (Wang et al., 2015; Zhang et al., 2015), vegetables and fruits (Campos et al., 2014; Wu et al., 2014), crops (Wei et al., 2013; Ling et al., 2014), grasses (Yang et al., 2015; Zhuang et al., 2015), and some wild plants grown in special habitats (Wang H. L. et al., 2014; Xiao et al., 2015). All these studies have proved the importance of reference gene screening and validation before gene expression analysis.

The tree peony (*Paeonia suffruticosa* Andr.) is an ornamental plant native to China with striking ornamental and medicinal value. It has a cultivation history going back more than 1600 years in China (Cheng and Li, 1998). Qualitative and quantitative analyses of anthocyanins have been conducted in *P. suffruticosa* (Zhang et al., 2007; Yin et al., 2012). A few genes involved in petal pigmentation have also been cloned in the tree peony (Zhou et al., 2013; Du et al., 2015; Shi et al., 2015). However, to date, there have been few studies on the comparison and selection of reference genes for qRT-PCR during flower development in tree peony. Only few studies focusing on the bud dormancy release (Zhang et al., 2011), various tissues (Liu et al., 2015), and different treatments in cut flowers (Wang et al., 2012) have been reported, showing that no gene has a constant expression profile under all developmental or experimental conditions.

To normalize the gene expression level during flower development in different cultivars of tree peony, 10 candidate reference genes were selected based on the floral transcriptome datasets of tree peony and the stability of their expression level was evaluated. The results provided a list of reference genes suitable for the accurate quantification of gene expression during flower development.

## Materials and methods

### Plant materials

Tree peonies were grown in the germplasms nursery of Shanghai Chenshan Botanical Garden. Three cultivars (‘Feng Dan,’ ‘Xi Shi,’ and ‘Que Hao’) were used as the plant materials. For each cultivar, petal samples were collected from three different plants at six opening stages (February to April, 2015). Samples from 9 plants (3 plants per cultivar) at 6 different developmental stages were taken in 2015: February 17, March 1, March 8, March 15, April 9, and April 14. The 54 petal samples were immediately frozen in liquid nitrogen and stored at −80°C until RNA extraction.

### RNA extraction and cDNA synthesis

Total RNA was extracted using an E.Z.N.A.™ Plant RNA Kit-R6827 (Omega) following its manufacturer instructions. To avoid DNA contamination, the RNA samples were treated with an RNase-free DNase Set (Omega). The total RNA concentration, purity and integrity were determined using a NanoDrop2000c Spectrophotometer (Thermo Scientific, U.S.) and visually assessed via 1.5% agarose gel electrophoresis. The RNA samples with absorption ratios of A260/A280 between 1.9 and 2.1 and A260/A230 ratios above 2.0 were used for subsequent analyses. First-strand cDNA was synthesized using 1 μg total RNA with the PrimeScript RT Master Mix (Perfect Real Time; Takara, Japan) according to the manufacturer's protocol.

### Candidate reference genes selection and PCR primer design

An Illumina/Solexa library of tree peony was constructed in our preliminary study. A total of 15019 unigenes (NR database) were annotated with BLASTX (data was not shown). The indices of mean expression value (MV), standard deviation (SD), and coefficient of variation (CV) value of RPKM, were calculated using the method described by de Jonge et al. (2007) for mining candidate reference genes from transcriptome data. A total of 10 candidate genes were selected and assessed (Table 1).

**Table 1:** **Primer sequences and amplification characteristics for 10 candidate reference genes and 1 gene of interest**.

**Unigene**	**Gene symbol**	**Gene name**	**Accession number**	**Arabidopsis Homolog locus**	**Primer [5′−3′] Forward/reverse**	**Product Size (bp)**	**TM (°C)**	**E (%)**	**R^2^**
comp39032_c0_seq1	Actin	Actin	KU853024	AT3G27000.1	AAGGTGATGGAATGGCTGAC/CAAGAACTGCTCCTCCAAGG	264	60	96.48	0.997
comp72555_c2_seq1	α-TUB	α-tubulin	KU853025	AT1G50010.1	AAAACTGTTGGTGGAGGCGA/TCTGGGTGAAAGAGTTGGCG	152	60	95.71	0.999
comp78934_c0_seq1	β-TUB	β-tubulin	KU853026	AT5G23860.2	AGAACGCCGACGAGTGTATG/AGGGAAACGAAGGCAGCAAG	149	60	90.51	0.994
comp21853_c0_seq1	UBC	Ubiquitin conjugating enzyme	KU853027	AT3G46460.1	TACCCAAACAGCCCTCCAAC/AGCAGCTTCAATGTTTGCCG	234	59	98.82	0.994
comp72593_c0_seq1	GAPDH	Glyceraldehyde 3-phosphate dehydrogenase	KU853028	AT1G13440.1	CTGGAGTTTTCACCGACAAGG/CCAATGGGGCAAGACAGTTAG	186	60	92.78	0.998
comp21491_c1_seq1	PP2A	Protein phosphatase 2A	KU853029	AT1G10430.1	TGATTACTTGCCCCTCACAGCC/CATAACAGGTCGCACATTGGTC	150	60	91.46	0.995
comp62257_c0_seq1	TIP41	TIP41-like protein	KU853030	AT4G34270.1	ACGTTCCATTCTCACCTCCC/GCTTCCAACCAGTAAGAGCG	164	59	101.45	0.993
comp54677_c1_seq1	SAMS	s-adenosylmethionine synthetase	KU853031	AT4G01850.2	TCTTCCACCTCAACCCATCTG/TAAGCACCACTCCTGTCCAC	166	59	104.74	0.991
comp82277_c2_seq1	EF-1α	Elongation factor 1 alpha	KU853032	AT5G60390.2	TCCTGGGCATCGTGACTTTA/TTCATCATACCGAGCCTTTG	232	60	94.57	0.997
comp62008_c0_seq1	CYP	cyclophilin	KU853033	AT2G36130.1	GTTCTTCATTACCTTGGCACCT/TGACCGTTGACCGTAGTATCTT	161	58	99.23	0.995
comp63583_c0_seq1	F3H	flavanone 3-hydroxylase	KU853034	AT3G51240.1	GCTCTACCGCCTGATGACAAG/GTTACTCGCTTCCACCCCTCC	191	60	98.58	0.996

Unigenes were selected from the transcriptome of tree peony. The mean efficiency (E) of PCR amplification and the regression coefficient (R^2^) for each primer pair were calculated by the LightCycler 96 SW Software.

Primers were designed with Primer Premier 5.0 (Lalitha, 2004), using the following criteria: targeting the 3′-untranslated region, no hairpin/dimer/false priming/cross dimer structures, Tm = 55–60°C, 18–25 bases in length, GC content between 40 and 60%, and amplicon length from 100 to 300 bp. The designed primer sets were BLASTed against the local floral transcriptional data of tree peony to verify primer specificity. Amplification efficiency (E) was evaluated using a standard curve generated by qRT-PCR using a 5-fold dilution series. The primer specificity was judged by melting-curve analysis and agarose gel electrophoresis analysis.

### Real-time quantitative PCR

Real-time PCR reactions were carried out in 96-well plates with a LightCycler96 System (Roche, Switzerland) using SYBR Green I Master. The reaction mixture contained 10 μL of SYBR Green I Master, 2.0 μL of diluted cDNA (1:25), 0.4 μL of each of the forward and reverse primers (10 μM), and 7.2 μL of PCR-grade water in a final volume of 20 μL. The following reaction conditions were applied: 30 s at 95°C, 40 cycles of 10 s at 95°C and 30 s at 60°C, and a melting curve protocol (65–95°C with fluorescence measured every 0.5°C). Each reaction was performed in three biological replicates and three technical replicates. Three no-template controls (NTC) were used to monitor possible DNA contamination.

### Analysis of gene expression stability and validation of reference genes

Three software programs (GeNorm 3.5, BestKeeper and NormFinder) were used to rank the stability of the 10 selected reference genes in the study. Moreover, RefFinder (http://fulxie.0fees.us/?type=reference), a comprehensive web-based tool was used to integrate and rank comprehensively the tested candidate genes. The raw Ct values were converted into relative quantities using the formula: E^−ΔCt^ (ΔCt = Ct value of each sample—the minimum Ct value) before data entry (Ramakers et al., 2003). All three of the software programs were run based on the software manuals to select suitable reference genes. RefFinder generated the final overall ranking of tested reference genes based on the geometric mean of the weights of every gene calculating by each program.

To validate the reliability of the reference genes, the relative expression levels of *PsF3H* in three cultivars, a key gene involved in the anthocyanin biosynthesis, was analyzed using the two top-ranking reference genes and the least stable reference gene, as determined by RefFinder, alone or in combination (calculated by geometric mean) for data normalization. The qRT-PCR amplification conditions were the same as described above. The primer specificity of *PsF3H* was verified as described for reference genes (Table 1). The relative expression data was calculated according to the 2^−ΔΔCq^ method and presented as fold change (Livak and Schmittgen, 2001).

## Results

### Amplification specificity and efficiency

The primer sequences and amplicon characteristics of 10 candidate reference genes were summarized in Table 1. The primer specificities were assessed by melting-curves and agarose gel electrophoresis analysis, which showed only a single product of the expected size (Figure S1A) and a single peak melting curves (Figure S1B) for each primer set. The amplification efficiency (E) of each primer pair varied between 90.51% for β*-TUB* and 104.74% for *SAMS*, and the regression coefficient (R^2^) ranged from 0.991(*SAMS*) to 0.999 (α*-TUB*; Table 1). The amplicon size ranged from 100 to 264 bp.

To assess the expression stability of the reference genes in different samples, the transcript abundances of 10 candidate reference genes were presented as their mean Ct values (quantification cycles). The mean Ct values of these candidates varied from 20.9 ± 1.2 (*SAMS*) to 26.8 ± 1.0 (*ACTIN*) in different samples. Genes with greater SD of Ct values have more variable expression than those with lower SD. Of the 10 candidate genes, *UBC* showed the least variation in gene expression (23.7 ± 0.6), and the expression of *SAMS* was the most variable (20.9 ± 1.2). The candidate genes can be ranked as: *UBC* > *EF-1*α*/TIP41* > *PP2A/CYP* > *GAPDH* > α*-TUB/ ACTIN* > β*-TUB* > *SAMS* (Table S1) based on the standard deviation of the Ct values.

### Expression stability of the reference genes

**Figure 1:**
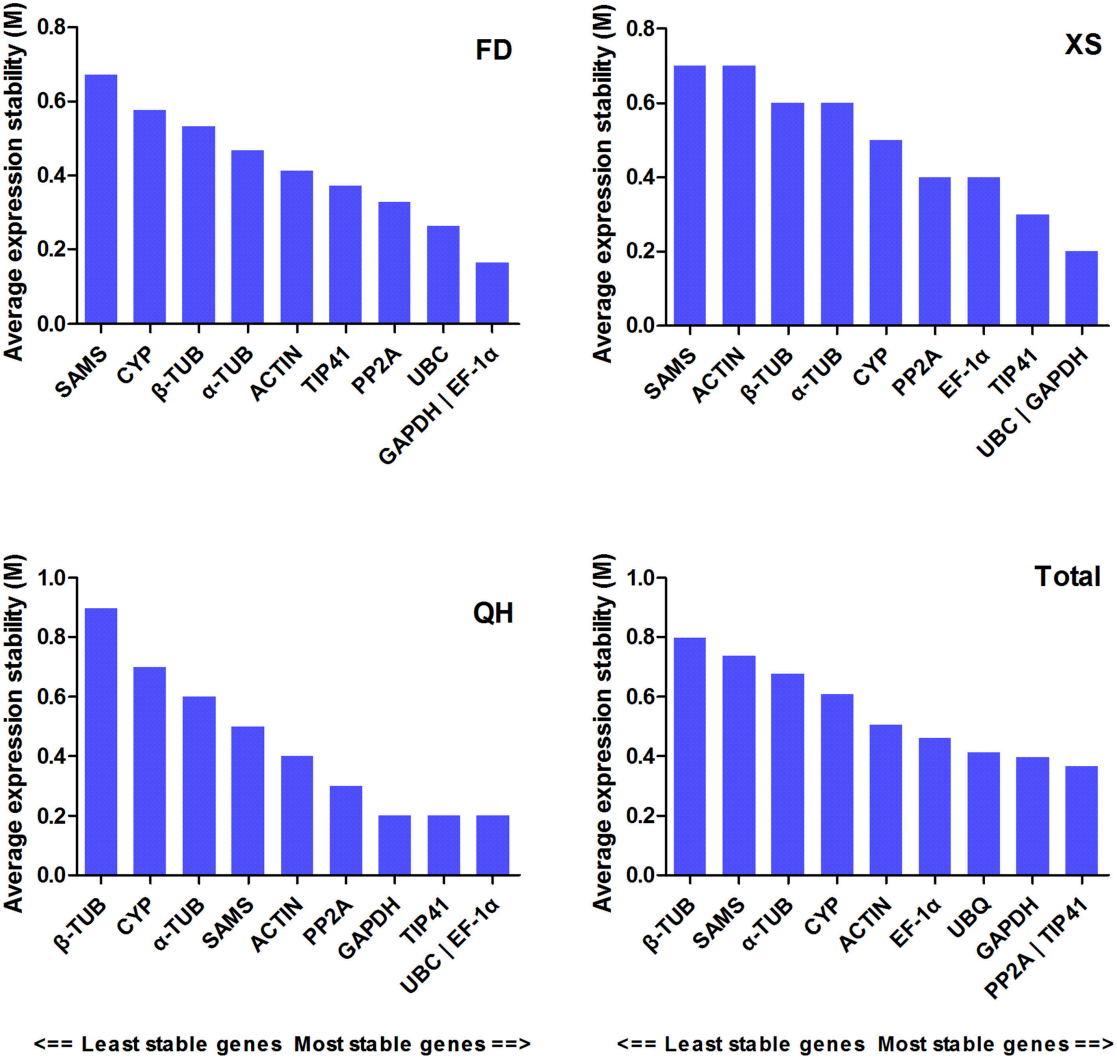
**The ranking order of reference genes. Expression stability values (M) of candidate reference genes calculated by GeNorm**. Lower *M*-value indicates more stable expression. FD, ‘Feng Dan’; XS, ‘Xi Shi’; QH, ‘Que Hao.’

**Figure 2:**
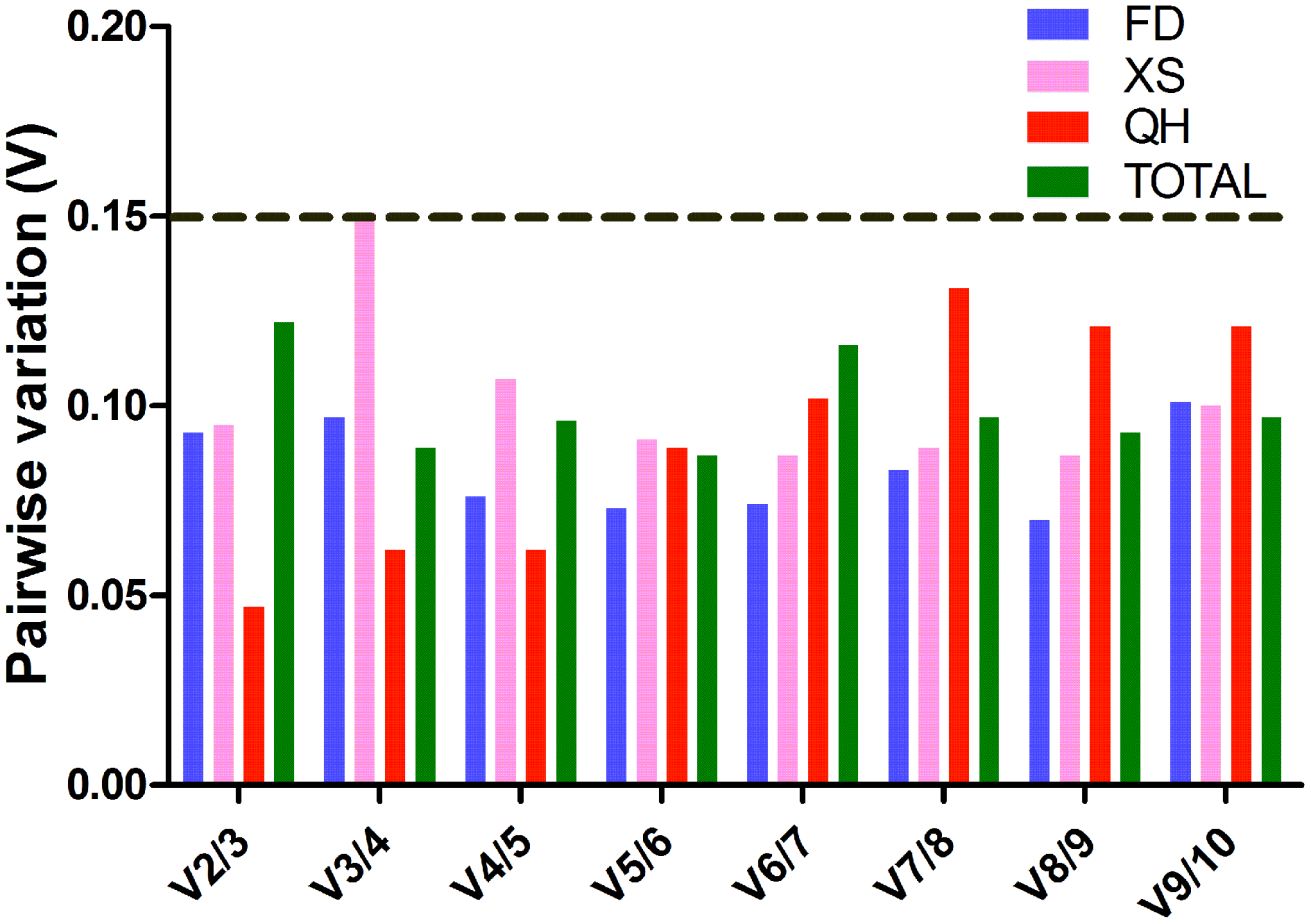
**Pairwise variation (V) of 10 candidate reference genes calculated by GeNorm**. The cutoff value to determine the optional number of reference genes for normalization is 0.15. FD, ‘Feng Dan’; XS, ‘Xi Shi’; QH, ‘Que Hao.’

Different software programs (GeNorm, NormFinder, BestKeeper, and RefFinder) were used to assess and rank the expression stability of the candidate genes. The average expression stability value (*M*-value) and the average pairwise variation of template normalization factor (V_n∕n+1_ value) were used to identify the most stable reference genes and obtain the required reference gene number in GeNorm. The *M*-value of a gene was inversely correlated with its expression stability, with the *M*-values less than 1.5 indicating stable expression (Hellemans et al., 2007). The cut-off value of 0.15 was used as a threshold of the V_n∕n+1_ value. If V_n∕n+1_ ≤ 0. 15, it is not necessary to introduce n+1 reference genes as the internal control. Figure 1 showed the ranking order of different candidate genes according to the *M*-value. For total samples, *PP2A* and *Tip41* were identified as the most stable genes, while β*-TUB* and *SAMS* were the least stable reference genes. As depicted in Figure 2, the optional number of reference genes for qRT-PCR data normalization was two in each cultivar. Taking the results of the most stable gene selection into account, the combinations *GAPDH/EF-1*α*, UBC/GAPDH*, and *UBC/EF-1*α were predicted to deliver the most reliable level of normalization for ‘Feng Dan,’ ‘Xi Shi,’ and ‘Que Hao,’ respectively.

NormFinder was used to determine the stability of reference genes based on inter- and intra-group variance in expression level. The stability value (SV) was calculated, with the lower stability value indicating the higher stability (Andersen et al., 2004). The results of NormFinder analysis were shown in Table 2. For total samples, *UBC* and *TIP41* were the most stable genes, while *SAMS* and β*-TUB* being the least stable. The top two most stably expressed genes were *GAPDH* and *UBC* in ‘Feng Dan,’ *GAPDH* and *TIP41* in ‘Xi Shi,’ and *TIP41* and *EF-1*α in ‘Que Hao,’ respectively. The ranking order was slightly different from that of GeNorm.

**Table 2:** **Expression stability of the candidate reference genes calculated by NormFinder**.

**Rank**	**FD**		**XS**		**QH**		**Total**	
	**Gene**	**Stability**	**Gene**	**Stability**	**Gene**	**Stability**	**Gene**	**Stability**
1	GAPDH	0.082	GAPDH	0.1	TIP41	0.0	UBC	0.219
2	UBC	0.131	TIP41	0.1	EF-1α	0.1	TIP41	0.222
3	EF-1α	0.149	UBC	0.2	UBC	0.1	GAPDH	0.243
4	PP2A	0.236	EF-1α	0.3	GAPDH	0.3	PP2A	0.326
5	TIP41	0.386	PP2A	0.4	PP2A	0.3	EF-1α	0.464
6	ACTIN	0.439	CYP	0.6	ACTIN	0.7	ACTIN	0.58
7	α-TUB	0.517	ACTIN	0.7	α-TUB	0.8	CYP	0.719
8	β-TUB	0.68	α-TUB	0.7	CYP	0.9	α-TUB	0.747
9	CYP	0.719	β-TUB	0.9	SAMS	1.0	β-TUB	0.918
10	SAMS	1.019	SAMS	1.0	β-TUB	1.2	SAMS	0.949

FD, ‘Feng Dan’; XS, ‘Xi Shi’; QH, ‘Que Hao.’

Coefficient of variance (CV) and the standard deviation (SD) were calculated by Bestkeeper analysis. The gene with the lowest CV and SD was considered to be the most stable reference gene (Chang et al., 2012). As shown in Table 3, *PP2A* and α*-TUB* were suggested to be the most stable genes in ‘Feng Dan.’ *UBC* and *GAPDH* had the lowest SD values in ‘Xi Shi,’ and *CYP* and *UBC* performed the best in ‘Que Hao.’

**Table 3:** **Expression stability of 10 candidate reference genes calculated by BestKeeper**.

**Rank**	**FD**		**XS**		**QH**		**Total**	
	**Gene**	**CV ± SD**	**Gene**	**CV ± SD**	**Gene**	**CV ± SD**	**Gene**	**CV ± SD**
1	PP2A	1.42 ± 0.34	UBC	0.60 ± 0.10	CYP	1.70 ± 0.50	UBC	2.03 ± 0.48
2	α-TUB	1.96 ± 0.45	GAPDH	1.00 ± 0.20	UBC	2.40 ± 0.60	EF-1α	2.22 ± 0.52
3	TIP41	1.75 ± 0.45	TIP41	1.00 ± 0.30	EF-1α	2.60 ± 0.60	TIP41	2.16 ± 0.56
4	GAPDH	2.25± 0.48	PP2A	1.50 ± 0.40	TIP41	2.30 ± 0.60	CYP	2.37 ± 0.62
5	UBC	2.28 ± 0.53	EF-1α	1.70 ± 0.40	α-TUB	3.10 ± 0.80	PP2A	2.64 ± 0.66
6	ACTIN	2.04± 0.53	ACTIN	2.10 ± 0.60	GAPDH	3.50 ± 0.80	GAPDH	3.34 ± 0.74
7	EF-1α	2.46 ± 0.58	α-TUB	2.40 ± 0.60	β-TUB	3.30 ± 0.80	α-TUB	3.55 ± 0.85
8	CYP	2.77 ± 0.71	CYP	2.20 ± 0.60	PP2A	3.30 ± 0.80	ACTIN	3.17 ± 0.85
9	SAMS	3.67 ± 0.76	β-TUB	2.60 ± 0.70	ACTIN	3.70 ± 1.00	β-TUB	3.53 ± 0.86
10	β-TUB	3.84 ± 0.90	SAMS	3.20 ± 0.70	SAMS	5.70 ± 1.20	SAMS	4.37 ± 0.91

FD, ‘Feng Dan’; XS, ‘Xi Shi’; QH, ‘Que Hao.’

### Comprehensive ranking of the reference genes by RefFinder

RefFinder was used to calculate the geometric mean of weights for the comprehensive ranking order recommended by geNorm, NormFinder, and BestKeeper. The comprehensive ranking order recommended by RefFinder was shown in Table 4. In total samples, the ranking order recommended by RefFinder was similar to those of NormFinder and GeNorm: *TIP41*> *UBC*> *GAPDH*> *PP2A*> *EF-1*α> *CYP*> *ACTIN*> α-*TUB*> β*-TUB* >*SAMS* (from the most stable to the least stable). *GAPDH* and *UBC* were ranked as the top two stable genes in ‘Feng Dan’ and ‘Xi Shi.’ *EF-1*α and *UBC* were the most stable genes in ‘Que Hao.’ β*-TUB* and *SAMS* were supposed to be the least stable genes in three cultivars.

**Table 4:** **Expression stability ranking of 10 candidate reference genes**.

**Method**	**1**	**2**	**3**	**4**	**5**	**6**	**7**	**8**	**9**	**10**
**RANKING ORDER IN FD (BETTER—GOOD—AVERAGE)**										
geNorm	GAPDH/EF-1α		UBC	PP2A	TIP41	ACTIN	α-TUB	β-TUB	CYP	SAMS
NormFinder	GAPDH	UBC	EF-1α	PP2A	TIP41	ACTIN	α-TUB	β-TUB	CYP	SAMS
Bestkeeper	PP2A	α-TUB	TIP41	GAPDH	UBC	ACTIN	EF-1α	CYP	SAMS	β-TUB
RefFinder	GAPDH	UBC	EF-1α	PP2A	TIP41	α-TUB	ACTIN	β-TUB	CYP	SAMS
**RANKING ORDER IN XS (BETTER—GOOD—AVERAGE)**										
geNorm	UBC/GAPDH		TIP41	EF-1α	PP2A	CYP	α-TUB	β-TUB	ACTIN	SAMS
NormFinder	GAPDH	TIP41	UBC	EF-1α	PP2A	CYP	ACTIN	α-TUB	β-TUB	SAMS
Bestkeeper	UBC	GAPDH	TIP41	PP2A	EF-1α	ACTIN	α-TUB	CYP	β-TUB	SAMS
RefFinder	GAPDH	UBC	TIP41	EF-1α	PP2A	CYP	ACTIN	α-TUB	β-TUB	SAMS
**RANKING ORDER IN QH (BETTER—GOOD—AVERAGE)**										
geNorm	UBC/EF-1α		TIP41	GAPDH	PP2A	ACTIN	SAMS	α-TUB	CYP	β-TUB
NormFinder	TIP41	EF-1α	UBC	GAPDH	PP2A	ACTIN	α-TUB	CYP	SAMS	β-TUB
Bestkeeper	CYP	UBC	EF-1α	TIP41	α-TUB	GAPDH	β-TUB	PP2A	ACTIN	SAMS
RefFinder	EF-1α	UBC	GAPDH	TIP41	CYP	PP2A	ACTIN	α-TUB	SAMS	β-TUB
**RANKING ORDER IN TOTAL SAMPLES (BETTER—GOOD—AVERAGE)**										
geNorm	PP2A/TIP41		GAPDH	UBC	EF-1α	ACTIN	CYP	α-TUB	SAMS	β-TUB
NormFinder	UBC	TIP41	GAPDH	PP2A	EF-1α	ACTIN	CYP	α-TUB	β-TUB	SAMS
Bestkeeper	UBC	EF-1α	TIP41	CYP	PP2A	GAPDH	α-TUB	ACTIN	β-TUB	SAMS
RefFinder	TIP41	UBC	GAPDH	PP2A	EF-1α	CYP	ACTIN	α-TUB	β-TUB	SAMS

FD, ‘Feng Dan’; XS, ‘Xi Shi’; QH, ‘Que Hao.’

### Validation of the reference genes

To assess the reliability of the selected candidate reference genes, the transcription levels of *PsF3H* were evaluated using different reference genes in three cultivars. The two most stable reference genes identified in each cultivar (*UBC and GAPDH* for ‘Feng Dan’ and ‘Xi Shi,’ *EF-1*α and *UBC* for ‘Que Hao’) and the least stable reference gene (*SAMS* or β*-TUB*) were used either singly or in combination in qRT-PCR analyses.

**Figure 3:**
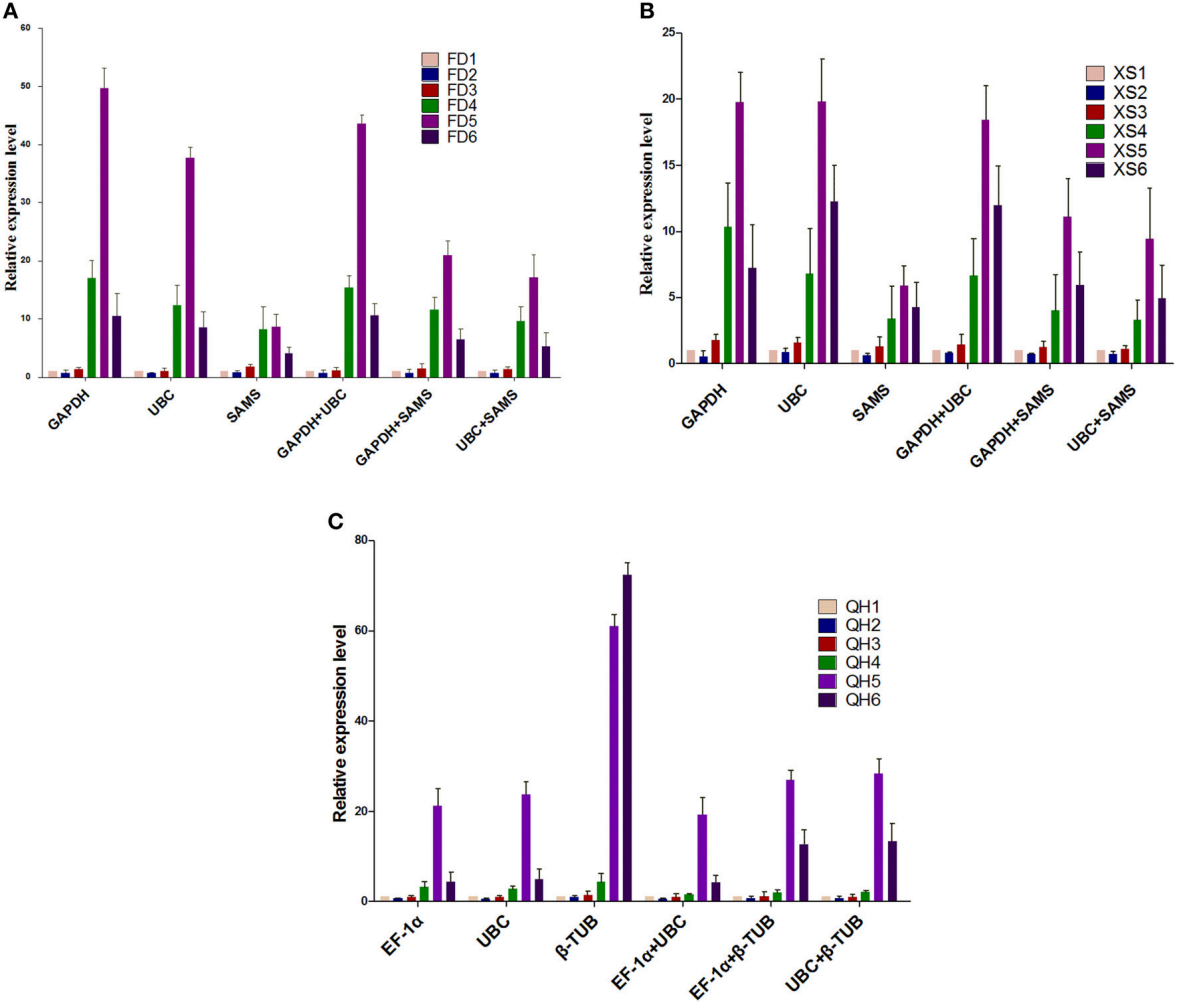
**Normalized expression level of *PsF3H* in different cultivars. (A)** Normalized expression level of Ps*F3H* in ‘Feng Dan’ (FD). Different reference genes (*GAPDH, UBC*, and *SAMS*) and gene combinations (*GAPDH* + *UBC, GAPDH* + *SAMS*, and *UBC* + *SAMS*) were used. **(B)** Normalized expression level of *PsF3H* in ‘Xi Shi’ (XS). The reference genes and gene combinations similar to ‘Feng Dan’ were used. **(C)** Normalized expression level of *PsF3H* in ‘Que Hao’ (QH). Individual genes: *EF-1*α, *UBC* and β*-TUB*; Combination genes: *EF-1*α + *UBC, EF-1*α + β*-TUB, UBC* + β*-TUB*.

Although the overall expression patterns of *PsF3H* were similar, differences were found when normalized to different reference genes. In ‘Feng Dan’ and ‘Xi Shi,’ the normalized expression levels of *PsF3H* using the least stable gene *SAMS* were dramatically decreased compared to the stable genes *GAPDH* and *UBC* (Figures 3A,B). Conversely, in ‘Feng Dan’, ‘Xi Shi’ and ‘Que Hao,’ the expression level normalized by β*-TUB* significantly increased compared to *EF-1*α and *UBC* (Figure 3C). When the combinations of *GAPDH/SMAS* and *UBC/SAMS* were used as internal control, the expression patterns of *PsF3H* were similar to that of *SAMS* (Figures 3A,B). When the combinations of *EF-1*α*/*β*-TUB* and *UBC*/β*-TUB* were used, the expression level of *PsF3H* at stage 6 was significantly overestimated. The combination of *GAPDH*/*UBC* was recommended to be the optimum pairs of reference gene for ‘Feng Dan’ and ‘Xi Shi,’ and *EF-1*α*/UBC* was the suitable pairs of reference gene for accurate normalization in ‘Que Hao.’

## Discussion

To ensure the accuracy of analysis, it is important to validate suitable reference genes for gene expression analysis using qRT-PCR. In this study, 10 reference genes were evaluated for the normalization of qRT-PCR in tree peony cultivars. For the cultivars ‘Feng Dan’ and ‘Xi Shi,’ the top two most stable reference genes, *GAPDH* and *UBC*, were similar, while *EF-1*α was identified as the most stable gene in ‘Que Hao.’ The expression stability of various reference genes differed across cultivars. Similar results were reported in strawberries and *Panax ginseng* (Galli et al., 2015; Wang and Lu, 2016). The diverse genetic background and biological process between cultivars probably affected the expression stability of reference genes. The results suggested that selection and validation of reliable reference genes for quantitative gene expression analysis was necessary not only for different species but also for different cultivars.

Among the candidate genes evaluated, *UBC* and *GAPDH* were the two most stable reference genes in both ‘Feng Dan’ and ‘Xi Shi’ cultivars. This result was consistent with previous studies in *Salicornia europaea* (Xiao et al., 2015), *Plukenetia volubilis* (Niu et al., 2015), and navel oranges (Wu et al., 2014). They seemed unstable for normalization at different development stages in petunia hybrid (Mallona et al., 2010). Both *ACTIN* and β*-TUB* were involved in basic cellular processes, and have long been considered suitable reference genes in numerous species (Jin et al., 2013; Zhang et al., 2015). However, in this study, β*-TUB* was identified as the least stable genes in all three cultivars.

With the development of RNA sequencing, increasing amounts of transcriptome data have been successfully utilized to screen reference genes for non-model plants (Demidenko et al., 2011; Xiao et al., 2015; Zhuang et al., 2015). It has been demonstrated that the newly discovered reference genes could perform better than traditional reference genes in *Brassica napus* (Yang et al., 2014) and tea (Hao et al., 2014). The results of this study also showed that, compared to classic reference genes, the newly discovered ones were more stably expressed in tree peony cultivars.

In conclusion, this study evaluated the expression stability of 10 candidate reference genes in three tree peony cultivars. *GAPDH*/*UBC* was identified as the optimum pair of reference genes in ‘Feng Dan’ and ‘Xi Shi.’ *EF-1*α*/UBC* was recommended to be the best combination for ‘Que Hao.’ The results provided useful information for selection and validation of reference genes for transcript normalization in gene expression studies in *P. suffruticosa*. In any case, a preliminary check on the accuracy of the best performing reference genes is requested for each qRT-PCR experiment.

## Author contributions

JL and JY conceived and designed the experiments. JL, JH, and YH performed the experiment and analyzed the data. JL wrote the paper. JY revised and approved the final manuscript.

### Conflict of interest statement

The authors declare that the research was conducted in the absence of any commercial or financial relationships that could be construed as a potential conflict of interest.
