# Supplementary material for: Selection of Reference Genes for Quantitative Real-Time PCR during Flower Development in Tree Peony (Paeonia suffruticosa Andr.)
Source: Front Plant Sci. 2016 Apr 21;7:516. doi: 10.3389/fpls.2016.00516 (PMC4838814; doi:10.3389/fpls.2016.00516)
Supplement: Supplementary file 1 [file Table1.docx]

**Table S1. Ct values of 10 tree peony candidate reference genes among tested samples.**

| Gene | Mean | Max | Min |
| --- | --- | --- | --- |
| SAMS | 20.9 ± 1.2 | 22.7 | 18.7 |
| GAPDH | 22.0 ± 0.9 | 23.5 | 20.4 |
| UBC | 23.7 ± 0.6 | 24.7 | 22.6 |
| EF-1α | 23.7 ± 0.7 | 25.0 | 22.5 |
| α-TUB | 23.9 ± 1.0 | 25.7 | 22.4 |
| β-TUB | 24.3± 1.1 | 26.6 | 21.9 |
| PP2A | 24.8 ± 0.8 | 26.0 | 23.6 |
| TIP41 | 26.1 ± 0.7 | 27.3 | 24.7 |
| CYP | 26.2 ± 0.8 | 27.3 | 24.2 |
| ACTIN | 26.8 ± 1.0 | 28.4 | 24.9 |
