# Supplementary material for: Selection of Reference Genes for Quantitative Real-Time PCR during Flower Development in Tree Peony (Paeonia suffruticosa Andr.)
Source: Front Plant Sci. 2016 Apr 21;7:516. doi: 10.3389/fpls.2016.00516 (PMC4838814; doi:10.3389/fpls.2016.00516)
Supplement: Supplementary file 2 [file Image1.PDF]

Selection of Reference Genes for Quantitative Real-time PCR during Flower Development in Tree Peony (*Paeonia suffruticosa*)

Jian Li[1, 2], Jigang Han[2], Yonghong Hu[2*] and Ji Yang[1*]

Supporting Information:

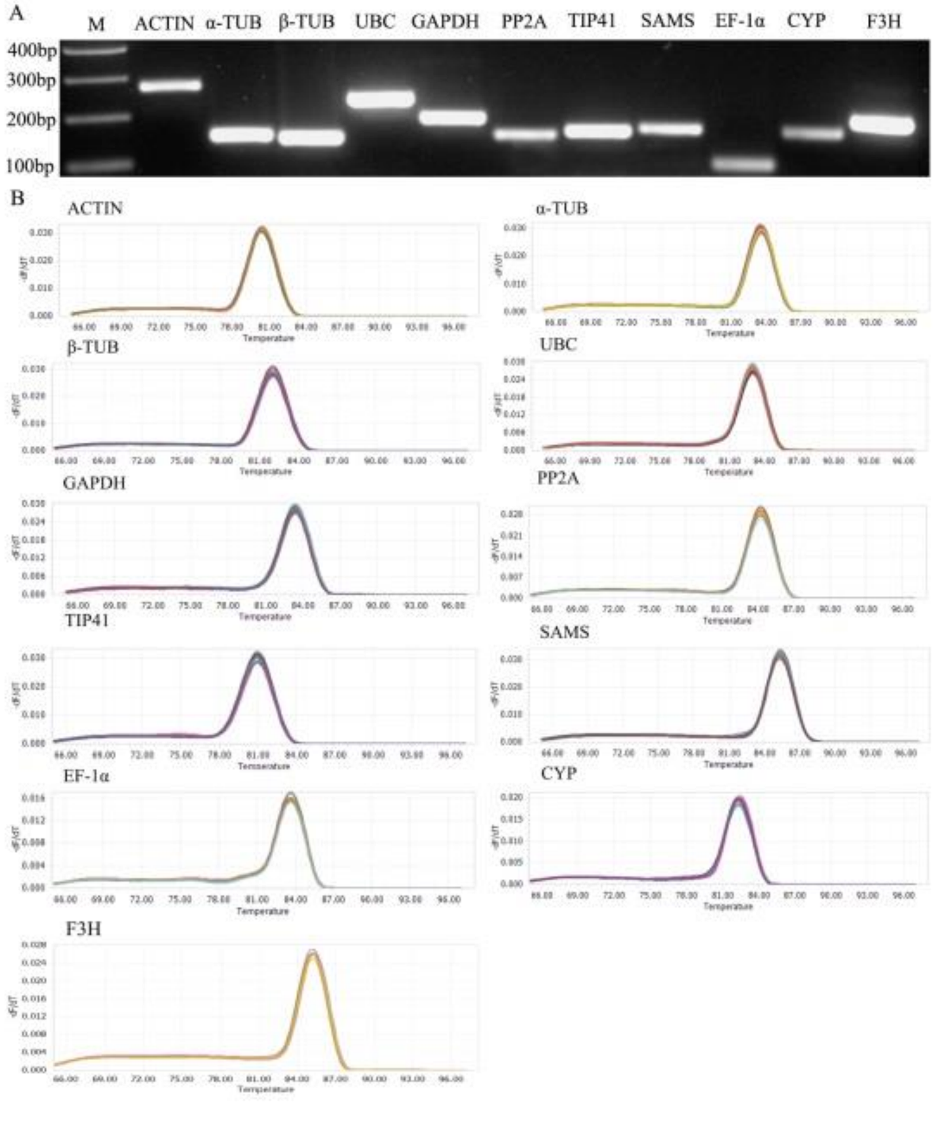

Figure S1. Agarose gel electrophoresis and melting curves of PCR products

(A) Agarose gel (2 %) electrophoresis showing amplification of a single PCR product of the expected size. M represents 100 bp DNA marker.

(B) Melting curves of 10 reference genes and 1 gene of interest showing single peaks.
